# Sexual and reproductive health needs assessment with deaf people in Ghana: Methodological challenges and ethical concerns

**DOI:** 10.4102/ajod.v2i1.55

**Published:** 2013-09-06

**Authors:** Wisdom K. Mprah

**Affiliations:** 1 Department of Disability and Human Development, University of Illinois at Chicago, United States of America

## Abstract

**Background:**

Deafness is a complex and multifaceted phenomenon. The different ways of perceiving and understanding deafness have practical implications for research with deaf people. Whilst the deaf community is not homogenous, it is generally distinct from the hearing population. Consequently, the appropriateness of applying research methods and informed concern processes designed for the hearing population in research with deaf people has been questioned.

**Objectives:**

This article reflected on some methodological challenges and ethical concerns arising from conducting a sexual and reproductive health needs assessment with deaf people in Ghana. The aim was to provide some perspectives on some of the challenges associated with doing research with deaf people.

**Method:**

The study was a two phase, sequential, mixed methods design, consisting of three focus groups to assist in the development of a survey and then the implementation of the survey for needs assessment data collection. The number of participants in the study was 179, consisting of 26 focus group participants: 7 executives of the Ghana National Association of the Deaf (GNAD), 10 male adults, and 9 female adults. There were 152 survey respondents (students, women and men) and one key informant. All participants, except the key informant, were deaf people.

**Results:**

The application of traditional research methods to studies involving deaf participants presents numerous methodological and ethical dilemmas associated mainly with deaf people’s unique cultural and linguistic characteristics.

**Conclusion:**

Research methods should not be taken as universal guidelines for conducting research in all settings because of differences in settings.

## Introduction

### Sexual and reproductive health and deaf people

Sexual and reproductive health (SRH) problems remain the leading causes of ill health and death worldwide, especially amongst women of reproductive age. The situation is worse in developing countries where millions of women suffer from long-term disabilities and premature death as a result of child-birth and pregnancy-related complications (Glasier *et al*. 2006).

Governments and other stakeholders are increasing their efforts to alleviate the consequences of poor SRH for individuals, families and society at large. A major landmark in this endeavour was the Unites Nations sponsored conference on population and development in Cairo in 1994, where a common course of action was taken to find solutions to the problem (United Nations Department of Public Information 1995). In Ghana, the formulation of policies (e.g. the Ghana Population Policy, the Adolescents Reproductive Health Policy and the National HIV/AIDS and STI Policy), research to identify groups at high risk, poverty reduction, and increasing access to information are key government strategies to address SRH problems (Ghana Statistical Service, Noguchi Memorial Institute for Medical Research & ORC Macro 2004; Hessburg *et al*. 2007).

However, people who are deaf and hard of hearing are unlikely to benefit from policies and programmes intended to address SRH problems. Negative perceptions about deafness and lack of societal understanding of their concerns have contributed to the neglect of deaf people in SRH policies and service delivery (World Health Organization [WHO] 2009). For example, available data suggest that deaf people are more likely to face difficulties utilising common sources of information than their hearing counterparts (Fedorowicz 2006; Groce, Yousafzai & Maas 2007; Heyederick 2006; Roberts 2006; Wilson & Monaghan 2006). They encounter communication barriers in the healthcare system because healthcare providers typically cannot communicate with them (Mottram 1999). In addition, healthcare providers often underestimate the difficulties of speech reading and overestimate deaf people’s ability to understand written notes (Margellos-Anast *et al*. 2005).

The few studies conducted on deaf people in Ghana indicated that they have limited access to mainstream information, and thus, have limited knowledge on SRH issues (Poku 2008; Tsiboe-Darko 2008). However, these studies do not provide comprehensive SRH data directly relevant to the deaf community. The purpose of this study therefore was to complement knowledge generated by these studies to provide a better explanation of the SRH needs of the deaf population in Ghana.

### Being deaf

Generally, deaf people are distinguished from the hearing population not only by their hearing loss, but by having distinct cultural and linguistic characteristics, which include a communication system that is different from the spoken language, as well as cultural values that are different from those found amongst hearing people (Sparrow 2005; Tucker 1998). In addition, whereas individualism is generally a dominant cultural feature in the hearing population (with some exception), collectivism is dominant amongst deaf people, and members of the deaf community often consider themselves as a close-knit and interconnected group (Ladd 2003).

In some western countries such as the USA, there are two main deaf cultural identities or perspectives: the medical, represented by a lowercase ‘d’, and the cultural or ethnic minority model, represented by a capitalised ‘D’. The distinction between d/Deaf formulation points to different perspectives of deaf individuals who are considered clinically deaf and those who are members of a linguistic-cultural group (Burch 2004; Padden & Humphries 2005). Deafness is perceived primarily in terms of the underlying medical pathology (Corker 1998; Tucker 1998). On the other hand, deaf people who subscribe to the cultural identity construction do not consider themselves as persons with disabilities and perceive deafness as a cultural phenomenon rather than a disability (Padden & Humphries 2005; Tucker 1998). The inability to hear, according to the cultural model, is essentially parallel to a hearing person’s ignorance of the sign language of the deaf community: a social disadvantage rather than a physical disability (Crouch 1997; Tucker 1998).

It should be noted that the distinction between ‘deaf’ and ‘Deaf’ identities is not so easy as it is a matter of perspective – there are deaf people who do not consider themselves part of Deaf culture and hearing people who do. Thus deafness is not a singular, monolithic entity; neither is it merely d/Deaf binary identities (Leigh 2009; Young & Hunt 2011). There are more substantial differences within the d/Deaf community than just the d/Deaf identities; for example, there are deaf people with varying degrees of hearing losses, fluency in sign language, literacy and level of integration in the community, all of whom have different life experiences and relate differently to their deafness (Leigh 2009; Young & Hunt 2011).

However, even though there are several ways of being deaf and several distinct deaf communities around the world, deaf people have many values in common, and these values are different from those of the hearing population. These differences have caused some people to question the appropriateness of applying traditional data collection methods, usually designed for the hearing population, in research with deaf people. Some researchers have suggested that research with deaf people should be considered cross-cultural. For example, Pollard (1992) argued that if it is acknowledged that there is the existence of a distinct deaf community and culture, which sometimes becomes the focus of research, then framing some research with deaf people as cross-cultural is appropriate.

This article is based on a SRH needs assessment with d/Deaf people in Ghana. The article discusses some of the critical issues and challenges involved in applying traditional research data collection methods and ethical principles in research involving deaf subjects. The intent is to provide insights into the possible challenges researchers working with d/Deaf subjects are likely to encounter if they ignore variability in the deaf community and presuppose a singular deaf identity. For the sake of convenience, the lowercase ‘deaf’ is used in this write-up.

## Methods of data collection and analysis

### Study design

The study was a participatory SRH needs assessment targeting only deaf people who were fluent in Ghanaian Sign Language (GSL) in Ghana. The study utilised a two-phase, sequential, mixed-methods design, consisting of three focus groups to assist in the development of a survey and then the implementation of the survey for needs assessment data collection. The focus groups allowed an in-depth exploration of themes to identify SRH issues that were important for the development of the quantitative (survey) instrument. The survey phase was conducted to document needs related to these themes within the deaf community.

Mixed methods research involves integrating two data collection techniques from two divergent research traditions in a single study. This approach presented opportunities for tapping the strengths of two methods, whilst at the same time compensating for their weaknesses. The qualitative component provides detailed perspectives or descriptions of processes, thus ensuring a better understanding of the phenomenon of interest, whilst the quantitative component highlights the potential causal mechanisms associated with a given outcome (Creswell & Plano Clark 2007; Curry, Nembhard & Bradle 2009).

Also, a mixed methods approach facilitates triangulation of data collected on the same issue, which often helps researchers develop a deeper understanding of the issue being investigated (Creswell & Plano Clark 2007). Triangulation allows the researcher to complement the differing strengths of quantitative and qualitative methods (Creswell & Plano Clark 2007).

Mixed methods research is therefore appropriate for investigating complex social problems that involve the needs of deprived communities. Issues concerning such communities are very complex and require research approaches that can contextualise and provide a comprehensive explanation of these issues. Data from multiple sources can help to provide a fuller understanding and to better interpret the results than relying on data from only one source. The complexity of SRH issues in Ghana presents similarly complex data collection and interpretation challenges, and thus makes mixed methods appropriate for this study.

### Population and sample

A total of 179 participants were recruited for the study, 26 of whom were focus group participants, 152 were survey respondents, and one person served as a key informant. All participants, except the key informant, were members of the deaf community and were considered well informed about issues in the community.

Sample size was a function of the available resources, time constraints and the difficulty of locating deaf people. The latter factor was an important limitation since deaf people do not form a homogenous population and do not normally reside in clustered localities. In addition, recruitment was limited to those with formal education who were fluent in GSL.

Participants were recruited from two communities in Ghana, namely Tamale, a city in the Northern Zone, and Accra, in the Southern Zone of Ghana. The intent in selecting these communities was to sample respondents with diverse characteristics so that views from people with different perspectives on the topic could be represented. Tamale and Accra represent the Northern and Southern sectors of the country, which reflect important differences in culture and socio-economic development. The Northern sector is generally poor and characterised by poorly developed infrastructure and harsh climatic conditions as compared with the Southern sector (Berry 1995; National Population Council 2000). Specific locations where participants were recruited from the two cities were a senior high school for the deaf, churches for the deaf and a centre for the deaf.

Efforts were made to ensure that women were equally represented since young girls have been found to be more at risk for SRH problems than boys (National Population Council 2000). Of the students recruited from the senior high school, 44 were female, although female students constituted only 93 of the 343 student population. In the study, respondents from Accra and Tamale (aged 22 years and above) are referred to as the ‘adult population’ and those from the senior high school for the deaf (aged 18–22 years) as ‘students’ or ‘adolescents’.

### Sampling strategy

The sampling procedure used for selecting participants for this study was purposive, targeting only persons considered knowledgeable of issues affecting the deaf community. Members were selected based on their knowledge of issues that affect the deaf community; they were considered opinion leaders in the deaf community. Those selected include current executives of GNAD, past executives of GNAD, and people serving on committees in churches for the deaf.

Whilst focus group participants were recruited from two churches for the deaf and a centre for the deaf in Accra, recruitment of survey respondents was conducted in a high school for the deaf and three churches for the deaf. These locations were selected in order to increase the likelihood of identifying deaf people who had formal education and knowledge of GSL. Recruitment was done through announcements that included information about the study and eligibility requirements. Informed consent was obtained from all participants before recruitment.

With the focus groups, prospective participants were contacted through text messages and emails. Written scripts of the recruitment announcements were developed in English and signed in GSL at introductory meetings. After contacting prospective participants, arrangements were made to meet the men and the women at two different locations to discuss the focus group procedures, their remuneration, and issues concerning their privacy and confidentiality. Ten out of the 12 men contacted agreed to participate, whilst 9 out of the 15 women contacted agreed to participate. Seven of the 10 GNAD executives agreed to participate. The key informant was recruited from one of the SRH centres.

Recruitment of survey respondents was conducted through announcements that included information about the study, eligibility requirements, and an invitation to volunteers to undergo screening and the informed consent process at predetermined dates and times. On the screening day, those who qualified to participate were asked to sign the informed consent forms. In the high school for the deaf, verbal permission was sought from the head of the school before recruitment began, and a notice was sent to teachers and students about the study. Based on advice from GNAD, one key informant was interviewed to seek his views on his experiences working with deaf people. He also helped to clarify information gathered from the focus groups and survey. The key informant had done a study on HIV and AIDS with the deaf community so he was familiar with that community.

### Criteria for exclusion

Participants comprised people who were deaf or hard of hearing, fluent in GSL, resident in Ghana and aged between 18 and 61 years. Lack of formal education was an exclusion criterion since formal education is required to use GSL. Communicating with this non-GSL group would have required learning the local language such persons developed to communicate within their communities – a serious logistical challenge since Ghana is a multilingual society. Users of GSL were more likely to have utilised or had attempted to utilise SRH information from education programmes that disseminated material through magazines, posters, online material and brochures. This category of deaf people were more likely to have better understanding and experiences to explain the challenges deaf people face when accessing SRH information and services.

## Data collection and analysis

### Focus groups

Three focus groups were conducted, (1) the executive group consisting of 7 executive members of GNAD, all of whom were men, (2) the adult male group with 10 members and (3) the adult female group with 9 members.

The focus group guide consisted of open-ended questions and elicited information on participants’ views concerning access to SRH services and information. Issues discussed were:

- sources of information
- knowledge of SRH problems in the deaf community
- SRH experiences and needs of deaf people
- ways to correct problems deaf people encounter when accessing information and services relating to SRH issues
- key related issues in the deaf community
- the role of GNAD in the provision of information and services relating to SRH issues.

Video tapes and audio recorders were used with participants’ permission to record proceedings in the focus group sessions. Whilst the men’s and the executives’ focus group sessions were conducted by a male research assistant, the women’s focus group session was conducted by a female research assistant. The researcher helped the assistants when probes were needed for clarification or when the discussions went off-topic. The men’s focus group session was the first to be conducted, followed by the women’s and then the executives’ sessions. Both the men’s and women’s focus group sessions were conducted on church premises whilst the executives’ was held at the GNAD head office.

The researcher and the research assistants were all native signers, so all focus group sessions were conducted in GSL. Being native signers and members of the deaf community in Ghana facilitated the establishment of rapport with the participants and created a comfortable environment to discuss issues relating to a sensitive topic in Ghana. In addition, resolving issues relating to the video recording of focus group participants and obtaining informed consent were made much easier by virtue of being members of the community.

The transcribed data from the three focus groups were analysed separately in order to differentiate the responses of the three categories of participants: leaders of the deaf community, male participants, and female participants. Focus group video tapes were converted to DVDs using Adobe Premiere Pro CS4 4.0.1 video software. Both the DVDs and the voice recordings were transcribed to text format.

The transcription of the data from the DVDs was done in two steps, namely ‘partial’ transcription and full transcription. The first step (‘partial’ transcription) involved viewing the DVDs from all the focus groups to identify and transcribe into text format concerns that were raised by participants. This was an abridged version of the discussions, consisting of only the group discussion material needed for the development of the survey. Since a verbatim transcription of the DVDs would require significant time and delay the development of the survey, an abbreviated procedure was employed. The second step was a ‘full’ transcription of the video tapes. The full transcription represented the data from the focus groups that were used to complement survey results from the final survey sample.

### Survey

Transcripts from the focus group video and audio, two existing surveys – the 2003 Ghana Demographic and Health Survey (GDHS) and a survey on SRH status amongst people with disabilities in Ghana – and two reports on adolescent reproductive health in Ghana were used to develop the survey.

The issues included in the survey were problematic areas drawn from the literature and additional concerns identified in the analysis of the focus groups transcripts. The final survey explored issues relating to factors influencing visits to SRH centres, organisations providing SRH services, SRH problems amongst deaf people, sources of information on SRH issues, level of knowledge on STIs and pregnancy, contraception knowledge and use, and importance and satisfaction ratings of SRH issues and services.

Based on advice from the GNAD, all the survey interviews were conducted in groups with the exception of the Tamale participants, who were interviewed individually. Each interview session involved gathering participants in a single room, distributing surveys and providing instructions. Research staff provided assistance and answered questions. Some of the items were written on blackboards and flip charts, which made it easier to explain items to all the respondents at the same time. The survey was conducted by the researcher and his two research assistants in GSL.

Basic descriptive statistics were used to analyse and summarise the survey data. Responses to the survey items were entered into a Statistical Package for the Social Sciences (SPSS) data file, and cross-tabulations and chi-square statistics were computed to compare response differences across age and gender groups.

### Findings

This study was undertaken to assess the SRH needs of the deaf community in Ghana, specifically those who use GSL, in order to find ways of improving access to SRH information and services. The findings from the study revealed that a wide range of factors limited access to vital SRH information and services to the deaf population in Ghana. For example, focus group findings indicated that deaf people encounter numerous obstacles when accessing SRH information and services. The obstacles are primarily associated with communication, but issues such as privacy and confidentiality at SRH centres, illiteracy amongst deaf people, ignorance of deaf people‘s needs, negative attitude towards deaf people, interpreters’ competence, and limited time for consultation have also contributed significantly in making health information and services inaccessible to the deaf community. Findings from the survey indicated that the level of knowledge on SRH issues amongst deaf people, particularly amongst adolescents, was low, possibly due to limited access to professional sources of information. This finding seems to support focus group findings about the difficulties deaf people face in accessing information from SRH centres.

Findings of the study were consistent with previous evaluations of general disability in Ghana and show many similarities between the deaf community in Ghana and the general population regarding knowledge and practice of SRH issues – knowledge of SRH issues is high but practice is low (Ghana Statistical Service, Noguchi Memorial Institute for Medical Research & ORC Macro 2004). The study findings also corroborated findings from other studies which indicate that health professionals were unable to communicate effectively with their deaf clients, with a negative impact on the quality of healthcare (Margellos-Anast *et al*. 2005; Mottram 1999).

Whilst the main aim of the study was to provide information to guide policy development, programme design and service provision for the deaf community in Ghana, it also has important methodological and ethical implications for conducting research with deaf people. These include the suitability of applying traditional data collection methods such as focus groups and surveys in research with deaf respondents and issues concerning obtaining informed consent and protecting the privacy and confidentiality of participants. These issues are discussed in the following sections.

## Methodological issues

### Transcribing focus group

The major methodological issue relating to the focus groups concerns transcribing and translating the video and audio recordings from sign language to word format. In the case of focus groups with hearing participants, audio recordings are transcribed verbatim, so that the ‘voices’ of participants are captured verbatim. With deaf participants, who use sign language, transcripts from video tapes may not represent the ‘voices’ of the participants because transcription will not be in sign language, but rather in English; as such, quotes will not be in the original language, which is sign language. This may affect the original meaning of the statements in sign language. The difficulty of transcribing sign language video tapes to text has been discussed by Ladd (2003). According to Ladd (2003:209) there are difficulties ‘… whenever the responses from Deaf participants required more than a “flat” English rendering of what was signed.’ This is more so in the current study because discussions in the focus groups were exclusively in the sign language, and there were difficulties making direct quotes from what has been signed.

### Conducting surveys

Issues relating to the survey concern the most appropriate approach to administer surveys with deaf respondents with diverse characteristics and the ability of respondents to understand survey items. In the present study, the survey respondents were interviewed in groups. Each interview session involved gathering participants in a single room, distributing surveys and providing instructions, whilst research staff went round to provide assistance and answered questions. The group interview sessions made it easier to administer the surveys because deaf people do not live in clustered locations, making it difficult to identify and recruit deaf subjects for individual interview sessions. However, this group interview strategy was not without difficulties. The main challenge was the difficult to manage the group interview sessions due to differences in the level of comprehension of survey items. Although some of the items were written on blackboards to make explanation easier – it made it possible to explain items to all the respondents at the same time without having to go round to assist each respondent who needed help – it was still difficult to handle the group effectively. The group interviews required more research assistants to assist respondents as almost every respondent wanted help because of the difficulty understanding survey items. There can be no universal rules on this issue; the best approach depends on many factors, including the time available for the study and the deaf subjects involved. Researchers should have adequate knowledge of the composition of their deaf subjects, and be flexible when designing and implementing research methods with deaf people.

Related to the above is respondents’ difficulty reading and understanding survey items. It has been noted that when surveys are written in respondents’ second language, their ability to understand and respond accurately to the survey items in order to reflect their real opinion or attitude may be inhibited (Turner 1993). As such, understanding survey items written in English can be challenging for deaf respondents whose English reading skill is low. Indeed, many respondents in the current study had difficulties understanding the survey items. The low reading skills of respondents is compounded by the sensitive nature of SRH issues in the Ghanaian culture, as well as the fact that GSL has fewer concepts related to the topic; there are concepts in the English language that do not exist in the GSL, such as ‘infection/contract’, ‘symptoms’, ‘UID’, ‘implant’, ‘female sterilisation’, and ‘male sterilisation’. These semantic differences and the general low literacy level amongst participants made it difficult to communicate these concepts. These problems may have resulted in the mistranslation of some concepts and may have made understanding survey items difficult for some participants. Researchers writing survey items should therefore take into consideration the linguistic barriers and communication preferences of the deaf participants and ensure that options with regard to communication exist for all participants in the study.

### Comparing research findings

Although comparing research findings drawn from one population with another population is a common strategy in contemporary research, comparisons between deaf people and hearing people may be inappropriate (Pollard 1992). Hence, the suitability of comparing study findings involving deaf subjects with those involving hearing subjects can be challenged because of differences in population and setting. This is particularly so in Ghana because deafness is viewed as a medical pathology and not a cultural phenomenon. Pollard (1992) warned of the need to make sure that, when differences are observed in comparing deaf and hearing people, such differences should not lead to conclusions that will be derogatory or demeaning to either group. Notwithstanding the diversity in the deaf community, there are significant differences between deaf and hearing people that should be taken into account when comparing research findings from one of the populations with the other.

## Ethical consideration

### Obtaining informed consent

Obtaining informed consent from research participants forms a vital part of the research process, and demands more than requesting signatures from participants. Investigators must ensure that potential subjects made informed decisions about whether or not they would want to participate in the research. This decision must be made freely, without coercion, and must be based on a clear understanding of what participation involves (Pedroni & Pimple 2001). This requires that research subjects are able to read and understand the consent forms in order to make informed decisions about their participation in the study.

There are therefore concerns about the appropriateness of using informed consent forms not written in the first language of the participants. Thus using informed consent forms written in English with deaf participants lead to some ethnical concerns. The current study used informed consent forms written in English; however, English is a second language for many deaf people. As a result, many of participants had difficulty understanding the informed consent forms. Consequently, informed consent in English may not be appropriate for some deaf subjects. Pollard (2002) observes that the low literacy level amongst deaf people can make it difficult for them to understand informed consent forms. Even the most accurate translation of the consent forms may still deprive some deaf people of vital information they need to make informed decisions about their involvement in a research study. Signing the informed consent form therefore does not necessarily imply that a deaf subject has made an informed decision to participate in the study (Pollard 2002). Overcoming these concerns require adopting options that are linguistically and culturally suitable for all deaf subjects.

### Confidentiality and anonymity of focus group participants

Researchers adopt various strategies to accurately capture feelings, experiences and reactions during focus group interactions. With hearing populations, the use of both video and audio recordings of focus group sessions is optional. In the case of focus groups with deaf participants, however, only video can be employed and sign interpreters may be needed to record and interpret what participants sign. If an investigator cannot do these tasks independently, then hiring video operators and sign language interpreters is necessary – people who are deeply involved in the discussion yet are not true participants. The presence of these ancillary staff compromises the anonymity and confidentiality of the data collection process (Pollard 2002). Indeed, in the present study participants in the focus groups were not comfortable with an ‘outsider’ doing the video tape recording because they thought their privacy would be compromised. Videos create additional privacy concerns since images are more readily identified than voice alone. Disguising the faces of participants in order to hide their identity is impossible without distorting or obscuring what was signed. And because the deaf community is closely knitted, extreme care must be taken in order to preserve the anonymity and confidentiality of participants (Ladd 2003; Pollard 2002). This is particularly so for users of GSL, since the majority attended the only senior high for the deaf in Ghana and so almost everyone knows everyone else.

## Conclusion

The purpose of the study was to identify the SRH needs of the deaf community in Ghana in order to identify concerns, and then make these visible for subsequent policy interventions. Since not much is known about the SRH status of the deaf community in Ghana, the study findings were also to complement existing knowledge on the topic so as to better explain the SRH experiences of the deaf population in Ghana.

The study findings indicated that the application of traditional research methods to studies involving deaf participants presents many methodological challenges, which include respondents’ inability to understand survey items, the best way of administering surveys to deaf respondents, and transcribing focus group video tapes. There were also ethical concerns relating to protecting the privacy and confidentially of participants and obtaining informed consent. In addition, there were challenges arising from misconceptions and the sensitive nature of SRH issues in the Ghanaian culture, the limited English reading skills of respondents, and the fact that Ghanaian sign language has fewer concepts relating to the topic of SRH. The study thus highlighted the methodological and ethical complexities in conducting SRH research with deaf people.

It should be noted that although the focus of the discussion was the deaf population in Ghana, the issues discussed extend beyond this population; the study has wider implications for research with deaf people in general. In order words, the study provides important background knowledge concerning the diversity of deaf identities and its implications, that is, how what it is to be deaf influences research and how it can complicate research with deaf people for many researchers, especially those unfamiliar with the nature of deaf identities. Finally, whilst efforts were made to avoid sweeping generalisations, it may happen, and therefore it is essential to acknowledge this limitation.

The significance of the study lies in alerting researchers about the importance of acknowledging the different ways of being deaf and how these influence research. Any research endeavour with deaf people that ignores the implications of diversity in the deaf community and the fluid deaf identity risks undermining the rigor and validity of the findings. Researchers should endeavour to establish good relationships with and acquire a good knowledge of the diverse groups in the deaf community. In this way, they will better understand the cultural values and the different categories of deaf identities.
